# Comparison of clinical outcomes of expansive open-door laminoplasty with unilateral or bilateral fixation and fusion for treating cervical spondylotic myelopathy: a multi-center prospective study

**DOI:** 10.1186/s12893-019-0583-8

**Published:** 2019-08-22

**Authors:** Nan Su, Qi Fei, Bing-Qiang Wang, Nan Kang, Qing-Ming Zhang, He-Hu Tang, Dong Li, Jin-Jun Li, Yong Yang

**Affiliations:** 1 Department of Orthopaedics, Beijing Friendship Hospital, No. 95 Yongan Road, Xicheng District, Beijing, 100050 China; 2 Department of Orthopaedics, Beijing Chao-Yang Hospital, Beijing, China; 3 Department of Orthopaedics, Beijing Xuan-Wu Hospital, Beijing, China; 4 Department of Orthopaedics, Beijing Bo-Ai Hospital, Beijing, China

**Keywords:** Cervical spondylotic myelopathy, Open-door laminoplasty, Fixation, Fusion

## Abstract

**Background:**

The present study evaluated the clinical outcomes and safety of expansive open-door laminoplasty, when securing with C4 – C6 lateral mass screw and fusion.

**Methods:**

A total of 110 patients with cervical spondylotic myelopathy (CSM) were enrolled. There were 88 male and 22 female, with mean age at 60.55 ± 10.95 years. All of the patients underwent expansive open-door laminoplasty with unilateral or bilateral C4–6 lateral mass screws fixation and fusion. Clinical data, including age, gender, operation-related information, pre- and post-operation Japanese Orthopedic Association (JOA) scores, and cervical curvatures were collected.

**Results:**

The mean follow-up time of the cohort was 13.61 ± 9.53 months. Among the 110 patients, 33 of them were allocated to Unilateral group, and 77 of them were in Bilateral group. The mean JOA score of the 110 patients before surgery was 10.07 ± 2.39, and the score was improved significantly to 12.85 ± 2.45 after surgery. There were no reported cases of neurological deterioration or symptom worsening. Patients in both the Unilateral group and Bilateral groups had significant improvement of JOA scores. Among all patients, the most frequently observed complications were axial symptoms (*n* = 7). The average preoperative cervical curvature among all patients was 15.17 ± 5.26, and the post-surgery curvature was 14.41 ± 4.29. Similar observations were found between Unilateral and Bilateral groups.

**Conclusion:**

The modified surgical approach provided satisfactory clinical outcome in patients with CSM. The unilateral and bilateral fixation appeared to provide similar outcomes, in terms of cervical curvature maintenance and improvement of clinical symptoms. However, the examination of the exact differences between the two fixation methods await further biomechanical studies.

## Introduction

Cervical spondylotic myelopathy (CSM) is a degenerative spinal disease that is most commonly found in people aged 55 years or older [1]. CSM can be caused by a direct compression of the cervical spinal cord or surrounding blood vessels. The symptoms of CSM ranged from numbness or weakness in upper extremity, loss of coordination, neck pain to quadriparesis or sphincter dysfunction. It was estimated that the CMS-related hospitalization was 4.04 per 100,000 person-years, and the number of CMS patients receiving surgical treatment has been increasing [2, 3]. In addition to the acquired degenerative processes, developmental canal stenosis (DCS) is a well-recognized predisposing factor for CSM development. Study showed that patients with CSM have a high incidence of DCS [4]. The congenital stenosis can be defined using X-ray images when the ratio of canal diameter to vertebral body diameter is less than 0.8 [5].

Surgery is often considered for treating CSM, aiming to decompress the cord with an expansion of the spinal canal. An anterior surgical approach is preferred when one to two levels are involved, while a posterior approach is considered when three or more levels are involved or when DCS is presence [1]. To perform laminoplasty, a satisfactory cervical lordosis is required for successful operation, as the cord may not drift posteriorly from the lesion with cervical kyphosis. However, there were some disadvantages of the posterior approach; for example, the lordosis angle may lost between 6.7° - 11.7°, or up to 35% [6, 7]. In order to prevent poor curvature of the cervical spine after surgery, many researchers modified the traditional surgical approach by persevering the nuchae muscle attachment [8], preserving or repairing the C2 semispinalis cervics [9], or preserving C7 spinous processes [10]. Some researchers also suggested the performance of C3–6 laminoplasty, instead of C3–7 [10, 11]. Nonetheless, the above surgical approaches are technically more challenge. The addition of fixation, such as Twinfix suture anchors [12] or titanium miniplates [13, 14], were used to avoid door closure.

Our institute has modified the posterior surgical approach, in which the expansive open-door laminoplasty was performed with the addition of short segment (C4–6) lateral mass screw fixation and fusion. This surgical approach achieved satisfactory clinical outcome in our previous single-center retrospective study [15]. To validate this surgical approach in different clinical settings, we performed a multi-center study. In this study, we also evaluated if there were any differences in efficacy between the unilateral and bilateral fixation. The clinical outcome and surgical related complications were reported here.

## Patients and methods

### Patient characteristics

This was a multi-center study, with a total of 110 patients enrolled. Four medical centers in China participated in this study, including Beijing Friendship hospital, Beijing Xuanwu hospital, Beijing Chaoyang hospital and Beijing Boai hospital. Ethic approvals were received from all institutes participated in this study. The patients were diagnosed with cervical spondylotic myelopathy (CSM) in combination with developmental cervical spinal canal stenosis between May 2014 and April 2017. CSM refers to a group of clinical symptoms that resulted from the compression of important structures around the cervical spine (e.g. nerve root, vertebral artery, sympathetic nerve). It is often caused by the degeneration of cervical intervertebral disc and surrounding bone and soft tissue. All of the patients fulfilled the eligibility criteria and provided informed consent. The main inclusion criteria included: (1) Diagnosis of CSM with developmental narrowing of the cervical spinal canal. Patients also presented symptoms of cervical spinal cord compression; (2) age 40 or above; (3) the stenosis spanned at three or more cervical segments. The main exclusion criteria included: (1) medical history of cervical spine fractures, dislocation, deformities, tumors, tuberculosis or other infection; (2) concomitant neurological diseases leading to pyramidal tract syndrome; (3) preoperative cervical kyphosis with C2–7 Cobb’s angle > 13°; (4) presence of severe osteoporosis or autoimmune diseases (e.g. rheumatoid arthritis, ankylosing spondylitis); (5) incomplete imaging data.

The functional status of patients with CSM was evaluated by Japanese Orthopaedic Association (JOA) score. Flexion-extension X-ray, cervical MRI, and CT examination were performed before surgery. All patients underwent posterior cervical expansive open-door laminoplasty, with short-segment (C4 to C6) unilateral or bilateral lateral mass screw and fusion. Patients had follow-up visits at 1-month, 3-month, 6-months, and 12-month post-surgery. JOA score assessment and X-ray imaging were performed during the follow-up visits.

### Surgical technique

Patients were not randomly assigned to unilateral or bilateral fixation, but in some cases, operators would perform bilateral fixation because of the patients’ condition (e.g. preoperative lordosis, poor cervical curvature, osteoporosis). The patient received general anesthesia and was placed in prone position on an operating table. The patient’s head was stabilized with a skull clamp. A posterior longitudinal incision was made in the midline of the neck to expose the C3–7 spinous process. Drilled holes were made at the lateral side of C4–6. Then, the interspinous ligaments at C2–3 and C7 – T1 were split, and the spinous processes were removed (with approximately 3 mm left). A hole in the base of the spinous process was made, and a needle was used to bring a silk suture thread through the hole. The outer cortex at the right side of C3–7 lamina base was removed using a burr, creating a door of about 3 mm width. The cortex at the left side of C3–7 lamina was removed to create a door of about 1.2 to 1.5 cm width. For patients in Unilateral group, three pairs of lateral mass screws were fixed at the right side of C4–6. For patients in Bilateral group, three pairs of lateral mass screws were fixed at C4–6 bilaterally. The resected spinous process was made into a matchstick-like autograft and was fixed securely in the laminar opening. The silk suture thread was tied to the screw to prevent closure of the laminoplasty. To close the wound, normal saline was used for copious irrigation of the wound. Retractors were removed and hemostasis was achieved. A standard layered closure was performed.

### Postoperative management

The drainage tube was removed 48 h after surgery, and antibiotics were administrated for 2 days. Methylprednisolone was used to reduce edema of spinal nerve roots (80 mg for 3 days). Low molecular weight heparin was used to prevent venous thromboembolism. Mobilization, and physical therapy started with the protection of Philadelphia neck collar after the drainage was removed. AP and lateral X-rays were obtained prior to patient discharge. Patients could discontinue the use of their collar after 3 months.

### Data collection and analyses

Complete medical records, including age, gender, duration of hospital stay, volume of blood loss, operation time, and intraoperative complications were collected. Preoperative and postoperative JOA scores were obtained (full score = 17 points). The postoperative improvement was calculated as previously described: (follow-up JOA score − preoperative JOA score)/(17 − preoperative JOA score) × 100% [15]. The X-ray images were analyzed, and cervical spine sagittal sequence (Cobb’s angle between C2 lateral endplate and C7 endplate) were obtained. All surgical related complications (e.g. screw loosening) were recorded during the analysis of X-ray images.

### Statistical analyses

Qualitative data were presented as percentage (%), and the measurement data were presented as mean ± standard deviation. For data that were normally distributed, Student’s t-test was used, while Wilcoxon two sample test was used for data that were not normally distributed. The pre- and post-operative JOA scores were analysed by paired t-test or Wilcoxon signed rank test. The post-operative JOA scores and curvatures between groups were compared using repeated measures ANOVA. All statistical analyses were performed using SAS9.3. Statistical significance was considered when *p* < 0.5.

## Results

One hundred ten patients were enrolled in this study, and 33 of them were allocated to Unilateral group and 77 of them were in Bilateral group. There were 88 male and 22 female, with mean age at 60.55 ± 10.95 years. The mean BMI was 25.34 ± 2.86 kg/m^2^. The mean follow-up time of the cohort was 13.61 ± 9.53 months, and a total of 32 patients had segmental instability. No significant differences were found in these characteristics between the Unilateral group and Bilateral group (Table 1).

**Table 1:** Clinical characteristics of patients

Parameters	Unilateral *n* = 33	Bilateral *n* = 77	All patients	*p*
Gender, n (%)				0.212
Male	24 (72.73)	64 (83.12)	88 (80.00)	
Female	9 (27.27)	13 (16.88)	22 (20.00)	
Age (yr), mean ± s.d.	58.73 ± 10.59	61.32 ± 11.07	60.55 ± 10.95	0.225
Minimum-Maximum	40–80	40–81	40–81	
BMI (kg/m^2^), mean ± s.d.	25.87 ± 2.89	25.09 ± 2.84	25.34 ± 2.86	0.205
Minimum-Maximum	20.7–35.49	18.37–32.87	18.37–35.49	
Follow-up time (month), mean ± s.d.	13.00 ± 7.52 3–36	13.87 ± 10.30 3–36	13.61 ± 9.53 3–36	0.791
Minimum-Maximum				
Segmental instability, n (%)	10 (30.30)	22 (28.57)	32 (29.09)	0.855

The intra- and post-operative conditions of patients were summarized in Table 2. The mean operation time was 150.58 ± 35.26 min, and the mean volume of blood loss was 331.37 ± 152.34 mL. The total drainage volume was 226.71 ± 130.01 mL. Patients stayed in hospital for approximately 17–18 days. Two cases of fat liquefaction were observed after surgery: one in the Unilateral group and one in the Bilateral group. These patients needed a longer time for wound healing, but no infection was observed. The above intra- and post-operative conditions were similar between the two groups; no significant differences were found. Overall, most of the patients had the bone autograft completely fused.

**Table 2:** Intraoperative and postoperative conditions

Parameters	Unilateral *n* = 33	Bilateral *n* = 77	All patients	*p*
Operation time (min), mean ± s.d.	141.82 ± 30.51	154.34 ± 36.65	150.58 ± 35.26	0.113
Intraoperative bleeding (ml), mean ± s.d.	333.06 ± 144.73	330.65 ± 156.4	331.37 ± 152.34	0.937
Total drainage (ml), mean ± s.d.	197.97 ± 89.22	239.03 ± 142.75	226.71 ± 130.01	0.085
Duration of hospital stay (day), mean ± s.d.	18.06 ± 7.40	17.75 ± 6.48	17.85 ± 6.74	0.768
Postoperative fat liquefaction, n (%)	1 (3.03)	1 (1.30)	2 (1.82)	0.512
Bone graft fusion, n (%)				
Completely fused	27 (84.38)	76 (98.70)	103 (94.50)	0.008
Partially fused	5 (15.63)	1 (1.30)	6 (5.50)	

Complications developed during the follow-up period were recorded (Table 3). There were no significant differences between Unilateral and Bilateral groups. Among all patients, the most frequently observed complications were axial symptoms (*n* = 7). The axial symptoms were mainly presented as pain or discomfort in neck and shoulders. Majority of the patients recovered within a year; some still experienced the discomfort, but their daily activates were not affected. There were 4 patients reported C5 palsy. C5 palsy was defined as the deterioration in power of the deltoid muscle by at least one grade. It may accompany with deterioration in power of the biceps, but not the deterioration in power of other upper arm muscles. Numbness or pain may also be presented in muscles innervated by C5. All patients with C5 palsy recovered within a year after surgery. Lastly, one case of caudal adjacent segment degeneration (ASD) and one case of internal fixation loosening were reported. ASD was defined as the presence of bone hyperplasia, intervertebral disc herniation, ligament hypertrophy, or cervical segment instability in adjacent segments. Overall, the complication rate was low.

**Table 3:** Complications after discharge from hospital

Complications during follow-up period	Unilateral *n* = 33	Bilateral *n* = 77	All patients	*p*
Segmental instability	1 (3.03)	4 (5.19)	5 (4.55)	1.000
C5 palsy	0 (0.00)	4 (5.19)	4 (3.64)	0.314
Axial symptoms	4 (12.12)	3 (3.90)	7 (6.36)	0.194
Caudal ASD	0 (0.00)	1 (1.30)	1 (0.91)	1.000
Loosening of internal fixation	0 (0.00)	1 (1.30)	1 (0.91)	1.000

*ASD* adjacent segment degeneration

The neurological recovery was measured by JOA score. The mean JOA score of the 110 patients before surgery was 10.07 ± 2.39, and the score was significantly improved to 12.85 ± 2.45 after surgery. The mean score increased to 14.66 ± 1.86 at 3 months after surgery (*p* < 0.05). There were no reported cases of neurological deterioration or symptom worsening. Patients in both of the Unilateral group and Bilateral groups had significant improvement of JOA scores at different time points (before discharge, 3-, 6-, 12-, and 24-month post-surgery), when compared to the baseline. No significant differences were found in JOA score between Unilateral group and Bilateral group (Table 4).

**Table 4:** Changes of JOA score before and after surgery

	Unilateral		Bilateral		All patients		*p*
	n	JOA score	n	JOA score	n	JOA score	
Before surgery	33	9.94 ± 2.34	77	10.13 ± 2.42	110	10.07 ± 2.39	0.683
Before discharge	33	12.73 ± 2.45 ^a^	77	12.91 ± 2.46 ^b^	110	12.85 ± 2.45 ^c^	0.687
3 months post-surgery	33	15.12 ± 1.63 ^a^	77	14.47 ± 1.93 ^b^	110	14.66 ± 1.86 ^c^	0.571
6 months post-surgery	9	15.56 ± 0.88 ^a^	18	15.39 ± 1.09 ^b^	27	15.44 ± 1.01 ^c^	0.712
12 months post-surgery	16	14.88 ± 2.16 ^a^	32	15.28 ± 1.25 ^b^	48	15.15 ± 1.6 ^c^	0.497
24 months post-surgery	6	15.17 ± 0.75 ^a^	8	14 ± 2.51 ^b^	14	14.5 ± 1.99 ^c^	0.081

^a^ compared to Unilateral before surgery, *p* < 0.05; ^b^ compared to Bilateral before surgery, *p* < 0.05; ^c^ compared to All patients before surgery, *p* < 0.05

**Fig. 1:**
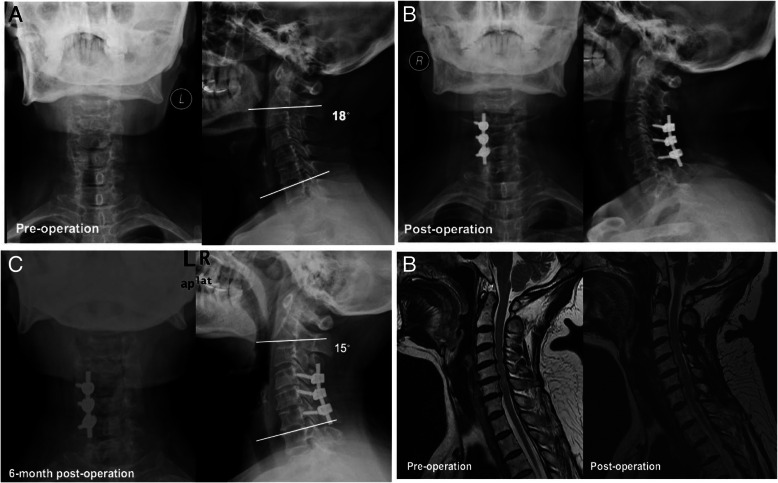
A representative case in Bilateral Group. **a** Cervical anterior-posterior X-ray image (*left panel*) and Cobb’s angle (*right panel*) before operation. **b** Postoperative anterior-posterior (*left panel*) and lateral (*right panel*) X-ray images. **c** 6-month postoperative anterior-posterior (*left panel*) and Cobb’s angle (*right panel*) X-ray images. **d** Preoperative MRI image showing the cord was compressed (*left panel*). Postoperative MRI image showing the compression of the cord was removed (*right panel*)

**Fig. 2:**
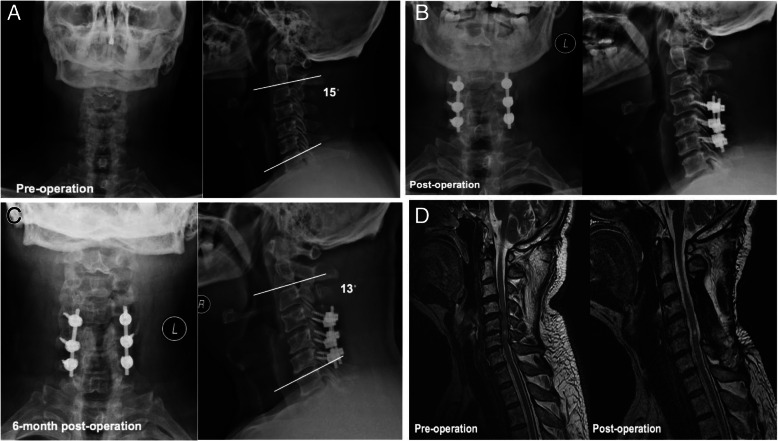
A representative case in Unilateral Group. **a** Cervical anterior-posterior X-ray image (*left panel*) and Cobb’s angle (*right panel*) before operation. **b** Postoperative anterior-posterior (*left panel*) and lateral (*right panel*) X-ray images. **c** 6-month postoperative anterior-posterior (*left panel*) and Cobb’s angle (*right panel*) X-ray images. **d** Preoperative MRI image showing the cord was compressed (*left panel*). Postoperative MRI image showing the compression of the cord was removed (*right panel*)

The X-ray images were analyzed to examine the cervical curvature (C2–7 Cobb’s angle) of patients. The average preoperative cervical curvature among all patients was 15.17 ± 5.26, and the post-surgery curvature was 14.41 ± 4.29 (Table 5). There was no significant difference between the pre- and post-surgery curvature. Similar observations were found in Unilateral and Bilateral groups. The Cobb’s angle was increased significantly at 6-, 12-, and 24-month post-surgery; however, it was noteworthy to mention that only a small portion of patients (*n* = 11 at 24-month) had X-ray images available for the analyses. Images of typical cases from Unilateral and Bilateral groups before and after surgery were shown in Figs. 1 and 2.

**Table 5:** Changes of curvature before and after surgery

	Unilateral		Bilateral		All patients		p*
	n	Curvature	n	Curvature	n	Curvature	
Before surgery	33	14.65 ± 5.16	77	15.39 ± 5.32	110	15.17 ± 5.26	0.445
Before discharge	33	14.82 ± 4.01	77	14.23 ± 4.42	110	14.41 ± 4.29	0.934
6 months post-surgery	10	15.56 ± 3.49 ^a^	19	15.78 ± 5.20	29	15.71 ± 4.62 ^c^	0.456
12 months post-surgery	17	19.78 ± 8.39 ^a^	32	21.76 ± 13.75 ^b^	49	21.07 ± 12.11 ^c^	0.635
24 months post-surgery	5	28.46 ± 3.82 ^a^	6	28.43 ± 7.56 ^b^	11	28.45 ± 5.87 ^c^	0.831

* Comparison between Unilateral and Bilateral; ^a^ compared to Unilateral before surgery, *p* < 0.05; ^b^ compared to Bilateral before surgery, *p* < 0.05; ^c^ compared to All patients before surgery, *p* < 0.05

## Discussion

The present study investigated the modified surgical approach for treatment of patients with CSM. We improved the traditional single-door decompression procedure by including the C4–6 lateral mass screw fixation and fusion. The use of lateral screws could maintain or in some cases correct the cervical lordosis. Also, the use of suture and bone autograft could prevent the fixation failure and to keep the door maintained in the open position. In our previous single-center retrospective study, this modified surgical approach promoted neurological recovery of patients: the JOA score increased from 9.14 ± 2.25 before surgery to 15.31 ± 1.73 after surgery, and the clinical symptoms were also significantly improved [15]. In this multi-center prospective study, the use of the modified surgical approach also provided satisfactory clinical outcome, indicating the reproducibility and effectiveness of the surgical method in treating CMS.

There has been controversy on the application of internal fixation for laminoplasty. Hyun et al. reported that the mean range of motion after cervical laminoplasty (including miniplate fixation) decreased by 10.1 ° ± 9.5° (31.66%) [16]. Another study also reported that the range of motion decreased 30.5, and 10.6% of patients experienced kyphosis after laminoplasty [17]. In view of these, we modified the single-door laminoplasty with the addition of short-segment C4–6 lateral fixation. This surgical approach retained the range of motion of the upper cervical vertebral (C1–3) and C7 – T1; in the meantime, incidences of postoperative neck stiffness and kyphosis were minimized. Our previous study showed that the use of this surgical approach could effectively maintain cervical lordosis after surgery [15]. In this study, although the cervical curvature decreased slightly after surgery, the change was not significant when compared to baseline (pre-surgery). Cervical lordosis was well maintained, and no cases of cervical kyphosis were reported.

Two surgical related complications were commonly seen in posterior cervical surgery, and they were axial pain and C5 palsy. The incidence rate of axial neck pain after laminoplasty was reported in 6–60% of patients [7, 18], while C5 palsy was reported in 4.8–11% of patients [19, 20]. In the current study, the incidence of axial pain was 6.36%, and the incidence of C5 palsy was 3.64%. The incident rates were similar to those reported, suggesting the use of short segment (C4–6) internal fixation would not increase the complication rate. Importantly, most patients with C5 palsy recovered within 1 year after surgery. Also, the axial pain was improved during follow-up visits. Overall, the use of short-segment lateral mass screw fixation would not increase neck stiffness, and the range of motion of the upper cervical vertebral (C1–3) was well maintained. The neck activities of patients, especially neck rotation, were not affected.

In this study, we also investigated the differences in clinical outcome between unilateral and bilateral fixation. It was appeared that both the unilateral and bilateral fixation had similar effects on improvement of clinical symptoms and maintenance of cervical curvature. There was no report of cervical kyphosis after surgery in both groups. However, it should be noted that some patients were not randomly assigned to unilateral or bilateral fixation. In some cases, operators would perform bilateral fixation because of the patients’ condition (e.g. preoperative lordosis, poor cervical curvature, osteoporosis). In addition, some patients experienced severe bleeding during surgery or had severe hyperplasia. In those cases, only the unilateral approach can be used. The limitation of adequacy to compare the outcomes between the two groups should be realized. In addition, we found inconsistency in the incidence of axial pain and C5 palsy between groups. For example, some centers had a higher incidence of axial pain in Unilateral group, while some centers had a higher incidence of axial pain in Bilateral group. This may due to the overall low incidences of complications, and the sample size was small. In some centers, there was only one case (or even none) of axial pain or C5 palsy. This may have certain impact on the results.

All of the lateral mass screws used in this study were fixed by free hand without using intraoperative imaging, like fluoroscopic control. A study reported that among the 1256 screws fixed using the free hand technique, the incidence of foramen transversarium violation was 0.876% (mostly in C6), and the incidence of facet violation was 1.433% [21]. Other studies reported that the poor surgical technique might cause penetration of the screw into the central portion of the transverse foramen, vertebral artery injury, or even brain stem infraction [22, 23]. Other screw fixation associated complications include nerve irritation, fracture of lateral mass, and screw pull-out [24]. In this study, there were no cases of vertebra artery, nerve root, or spinal cord injury. This may be related to the lateral mass screws selected in this study, which were short with length at 14 mm. Also, we did not require to have cortex fixation. There were no cases of screw displacement or loosening after surgery, suggesting the safety and effectiveness of the internal fixation.

## Conclusion

The current study showed that the posterior open-door laminoplasty in combination with C4–6 short segment lateral mass screw fixation and fusion provided satisfactory clinical outcome in patients with CSM. The short-segment internal fixation could help to maintain the cervical lordosis. Lastly, the unilateral and bilateral fixation appeared to provide similar outcomes, in terms of cervical curvature maintenance and improvement of clinical symptoms. However, it is worth to note that some patients were not randomly assigned to unilateral or bilateral fixation, because of the patients’ comorbid condition. The examination of the exact differences between the two fixation methods await further biomechanical studies.

## Data Availability

The datasets used and analyzed during the current study are available from the corresponding author on reasonable request.

## Abbreviations

CSM: Cervical spondylotic myelopathy
DCS: Developmental canal stenosis
JOA: Japanese Orthopedic Association
